# The effects of super-flux (high performance) dialyzer on the plasma glycosylated pro-B-type natriuretic peptide (proBNP) and glycosylated N-terminal proBNP in end-stage renal disease patients on dialysis

**DOI:** 10.1186/2050-6511-14-S1-P47

**Published:** 2013-08-29

**Authors:** Yasuaki Nakagawa, Toshio Nishikimi, Naoto Minamino, Chinatsu Yamada, Kazuhiro Nakao, Takeya Minami, Yoshihiro Kuwabara, Hideyuki Kinoshita, Shinji Yasuno, Kenji Ueshima, Koichiro Kuwahara, Kenji Kangawa, Kazuwa Nakao

**Affiliations:** 1 Department of Medicine and Clinical Science, Kyoto Univ. Graduate School of Medicine, Japan; 2 Department of EBM Research, Kyoto University Hospital, Institute for Advancement of Clinical and Translational Science, Japan; 3 Research Institute National Cardiovascular Research Center, Fujii Hospital, Japan; 4 Department of Cardiology, Fujii Hospital, Japan

## Background

The current BNP immunoassay cross-reacts with glycosylated proBNP, and the NT-proBNP assay underestimates glycosylated NT-proBNP. In addition, the recently developed high performance dialyzer removes medium-sized molecular solutes such as β2-microgloburin. We therefore investigated the effects of high performance dialysis on measured levels of glycosylated proBNP, glycosylated NT-proBNP and other BNP-related peptides in end-stage renal disease (ESRD) patients on hemodialysis.

## Method

We used our newly developed immunoassay to measure plasma total BNP, proBNP and mature BNP in 36 ESRD patients before and after hemodialysis. Plasma glycosylated NT-proBNP and nonglycosylated NT-proBNP were measured using Elecsys II after treatment with the deglycosylating enzymes neuramoinidase and *O*-glycosydase. We also measured plasma ANP and cGMP using radioimmunoassays.

## Results

Total BNP (-38.9%), proBNP (-29.7%), mature BNP (-54%), glycosylated NT-proBNP(-45.5%), nonglycosylated NT-proBNP(-53.4%), ANP(-50.4%) and cGMP(-72.1%) were all significantly reduced after hemodialysis. The relative magnitudes of the reductions did not correlate with any indices of plasma volume, but instead appeared to be molecular weight dependent. The proBNP/total BNP and glycosylated NT-proBNP/nonglycosylated NT-proBNP ratios were increased after hemodialysis. The proBNP/total BNP ratio correlated positively with hemodialysis vintage and negatively with left atrial diameter and systolic blood pressure, whereas glycosylated/nonglycosylated NT-proBNP ratios correlated positively with parathyroid hormone levels.

## Conclusion

These results suggest that plasma BNP and its related peptides measured immediately after hemodialysis may not be good indices of body fluid status in ESRD patients undergoing hemodialysis using a high performance dialyzer. ProBNP/total BNP may be influenced by hemodialysis vintage, cardiac afterload and diastolic function.
